# HMGA1 promotes metastatic processes in basal-like breast cancer regulating EMT and stemness

**DOI:** 10.18632/oncotarget.1136

**Published:** 2013-07-09

**Authors:** Silvia Pegoraro, Gloria Ros, Silvano Piazza, Roberta Sommaggio, Yari Ciani, Antonio Rosato, Riccardo Sgarra, Giannino Del Sal, Guidalberto Manfioletti

**Affiliations:** ^1^ Dipartimento di Scienze della Vita, Università degli Studi di Trieste, Trieste, Italy; ^2^ Laboratorio Nazionale CIB, (LNCIB), Area Science Park, Trieste, Italy; ^3^ Department of Surgery, Oncology and Gastroenterology, University of Padova, Padova, Italy; ^4^ Istituto Oncologico Veneto IRCCS, Padova, Italy

**Keywords:** Epithelial to mesenchymal transition, stemness, HMGA1, breast cancer, invasion, metastasis, gene-signature

## Abstract

Breast cancer is a heterogeneous disease that progresses to the critical hallmark of metastasis. In the present study, we show that the High Mobility Group A1 (HMGA1) protein plays a fundamental role in this process in basal-like breast cancer subtype. HMGA1 knockdown induces the mesenchymal to epithelial transition and dramatically decreases stemness and self-renewal. Notably, HMGA1 depletion in basal-like breast cancer cell lines reduced migration and invasion *in vitro* and the formation of metastases *in vivo*. Mechanistically, HMGA1 activated stemness and key migration-associated genes which were linked to the Wnt/beta-catenin, Notch and Pin1/mutant p53 signalling pathways. Moreover, we identified a specific HMGA1 gene expression signature that was activated in a large subset of human primary breast tumours and was associated with poor prognosis. Taken together, these data provide new insights into the role of HMGA1 in the acquisition of aggressive features in breast cancer.

## INTRODUCTION

Breast cancer is the most common cancer in women and a leading cause of cancer mortality in Western countries [1]. Based on microarray analyses, this tumour type has been classified into distinct molecular subtypes, including normal-like, luminal A and B, HER2+ and basal-like [2, 3]. The basal-like subtype is characterised by resistance to chemotherapy, frequent expression of cancer stem cell markers and unfavourable prognoses due to the highly metastatic phenotype [4, 5].

Recent advances in our understanding of the biology underlying tumour progression and metastasis suggest that cancer stem cells (CSCs) are important elements that promote the progression of primary tumours to metastatic disease [6]. In addition, the epithelial to mesenchymal transition (EMT), a key developmental programme that is often activated during cancer invasion and metastasis, has been associated with the acquisition of CSC characteristics [7, 8].

High Mobility Group A (HMGA) proteins are architectural factors that constitute critical hubs in the chromatin network. HMGA proteins, including HMGA1 (with the splice variants HMGA1a and HMGA1b) and the highly related HMGA2, bind AT-rich DNA stretches, forming stereospecific, multiprotein complexes called “enhanceosomes” on the promoter/enhancer regions of genes that regulate gene transcription [9, 10].

HMGA family members play important roles in stem cell self-renewal, proliferation and differentiation [11]. In normal cells, the expression of HMGA proteins is restricted to embryogenesis and, with few exceptions, is very low or almost absent in normal adult cells [12]. However, HMGA proteins are re-expressed at high levels in transformed cells, representing a general feature of human malignancies. Several studies have reported that HMGA1 expression is elevated in a variety of human cancers, including carcinomas derived from thyroid, prostate, colon and breast tissues [13].

In recent years, studies have demonstrated a causal role of the HMGA1 protein in promoting a transformed phenotype [14-19]. Moreover, the presence of the HMGA1 protein has been correlated with a higher cancer grade in mammary epithelial cancer [20, 21], suggesting that HMGA1 may be a key player in sustaining breast cancer. In this study, we demonstrate that HMGA1, by cooperating with the Wnt/beta-catenin and Pin1/mutant p53 signalling pathways, is fundamental in sustaining stem cell and metastatic properties in basal-like breast cancer subtype. To further evaluate the clinical significance of HMGA1 in breast cancer, we combined microarray analyses with clinical information regarding primary breast tumours and found that HMGA1 regulates a set of genes that may potentially be used as an independent predictor of poor clinical outcomes in breast cancer.

## RESULTS

### HMGA1 expression in primary breast tumours

**Figure 1:**
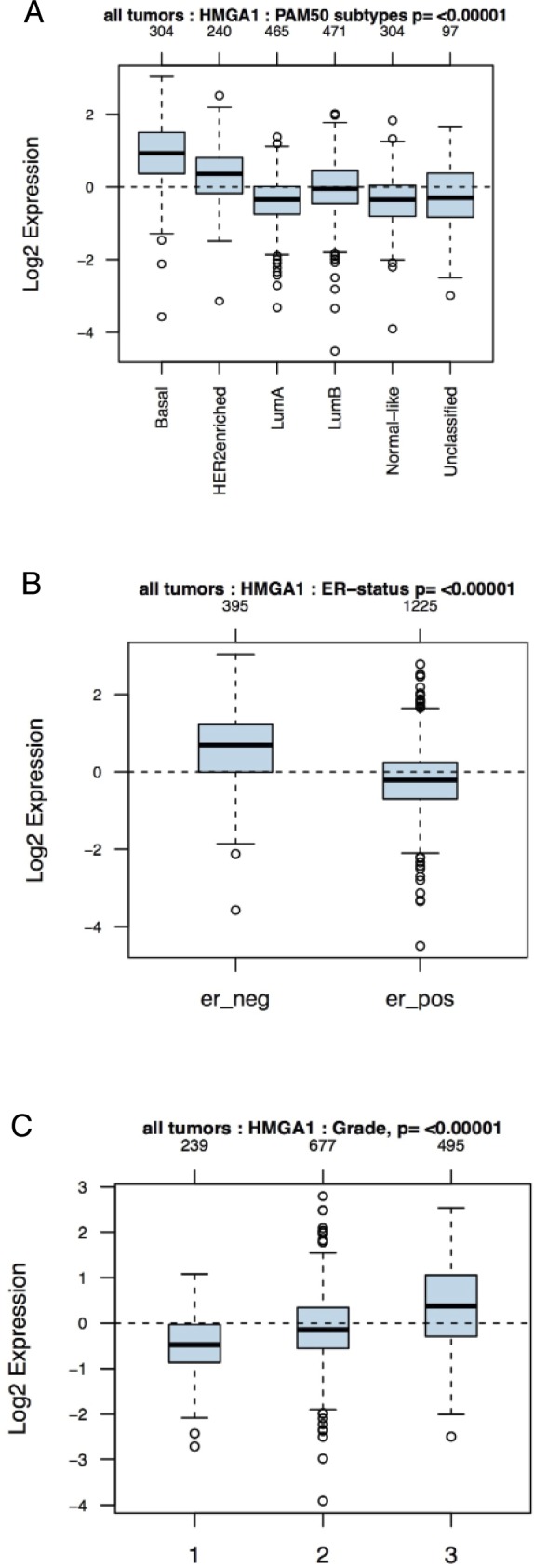
Association of HMGA1 mRNA Levels with Subtype and Tumour Grade in Human Breast Cancers Boxplots of the distribution of the gene expression intensities of HMGA1 mRNA across different (A) breast cancer subtypes, (B) ER-statuses and (C) tumour grades.

A previous study has shown that HMGA1 is overexpressed in 60% of sporadic ductal carcinomas [20], but it is unclear whether HMGA1 is enriched in a particular molecular subtype. To elucidate the importance of HMGA1 in breast cancer, we compared the abundance of HMGA1 mRNA with the genetic tumour subtype and histological grade, which are important indicators of breast cancer prognosis [2, 3], by performing a bioinformatic analysis of a primary breast cancer public microarray data collection (1881 different samples). HMGA1 mRNA levels were higher in the basal-like and HER2+ subtypes than the luminal A and B and normal-like subtypes (Fig. 1A). Moreover, as both the basal-like and HER2+ subtypes are oestrogen receptor-negative breast cancer subtypes, we found a strong association between HMGA1 mRNA expression and the oestrogen receptor-negative subtype (Fig. 1B). Finally, higher HMGA1 mRNA expression was associated with more advanced tumour grade (Fig. 1C). These results were in agreement with previously described immunohistochemistry data for the HMGA1 protein [22].

### HMGA1 silencing reverts the breast cancer cell transformation phenotype

**Figure 2:**
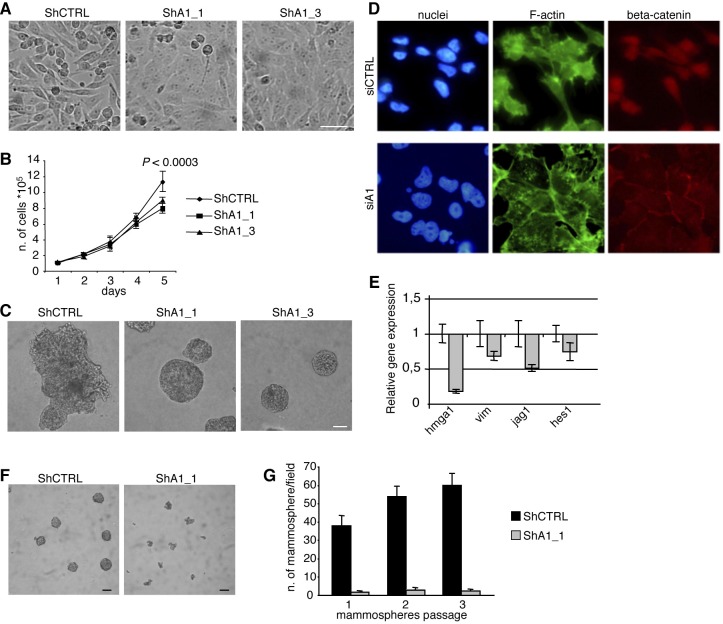
HMGA1 Depletion Induces Phenotypic Changes in the MDA-MB-231 Breast Cancer Cell Line (A) Representative pictures illustrating that HMGA1 depletion (ShA1_1 and ShA1_3) induces morphological changes in MDA-MB-231 cells. ShA1_1 and ShA1_3 are different clones obtained using two shRNA molecules targeting a region in the 3'UTR and the coding sequence of HMGA1, respectively, to avoid potential off-target effects. ShCTRL was obtained using a control shRNA. The scale bar represents 50 μm. (B) Proliferation curves. The data are represented as the means ± SD (n=3). The presented data were obtained from two independent clones for MDA-MB-231 ShCTRL, ShA1_1 and ShA1_3. (C) Phase contrast pictures of 3D growth after 11 days in culture. The scale bar represents 50 μm. (D) Representative images of cells that have been transfected with control (siCTRL) and HMGA1 (siA1) siRNA and stained with phalloidin (green) to visualise F-actin. Immunofluorescence for beta-catenin (red). Nuclei are stained with Hoechst (blue). (E) The down-regulation of selected genes after HMGA1 silencing (gray bar) was measured using real-time PCR. Expression was normalised to the levels in MDA-MB-231 cells that had been transfected with control siRNA. GAPDH was used as an internal control. The data are represented as the means ± SD (n=3). (F) Representative phase contrast pictures of mammosphere growth after 12 days in culture. The scale bar represents 100 m. (G) *In vitro* quantification of mammospheres that were formed by the cells described in (F). In (A) and (C), representative clones for ShCTRL, ShA1_1 and ShA1_3 are shown.

To determine whether HMGA1 signalling is an important oncogenic event in basal-like breast tumours, we generated an inducible cellular system for HMGA1 silencing based on short hairpin RNA (shRNA) in the oestrogen receptor-negative basal-like human breast cancer cell line MDA-MB-231 and checked for HMGA1 down-regulation after induction (Supplementary Fig. S1). Strikingly, we observed significant morphological alterations to a more differentiated phenotype after HMGA1 depletion. HMGA1-silenced cells (ShA1_1 and ShA1_3) displayed a flattened and polygonal morphology and grew as a highly ordered monolayer sheet, whereas control cells (ShCTRL) maintained a spindle-like fibroblastic phenotype (Fig. 2A). Consistent with these observations, we found that the proliferation rate of HMGA1-silenced cells decreased when the cells reached confluence (Fig. 2B), suggesting that the reversion to an epithelial phenotype may be accompanied by the re-acquisition of cell-cell contact inhibition, altering the growth capacity of MDA-MB-231 cells. To gain further insight into the potential role of HMGA1 in differentiation, we analysed the effects of HMGA1 depletion in a 3D assay growing cells on Matrigel [23]. Interestingly, in HMGA1-depleted cells, the disorganised morphology changed to acini-like spheroids with hollow lumens typical of non-malignant breast epithelial cells (Fig. 2C), confirming that HMGA1 may be involved in the tumour cell differentiation process.

Because the EMT plays a central role in differentiation and tumour initiation [24], we next investigated the impact of HMGA1 expression on the EMT. In HMGA1-depleted cells, we observed a reorganisation of the actin cytoskeleton in which F-actin was arranged in a cortical pattern and was accompanied by a drastic reduction of stress fibres (Fig. 2D) and down-regulation of the expression of mRNA encoding the mesenchymal marker vimentin (Fig. 2E). In addition, we observed regulation of EMT-inducing signalling pathways, including the Notch and Wnt/beta-catenin pathways. In fact, the expression of mRNAs encoding key players (such as Jag1 and Hes1) in the Notch pathway was down-regulated (Fig. 2E). Concomitantly, we observed the relocalisation of beta-catenin from the nuclei to cell-cell contacts (Fig. 2D) following HMGA1 depletion, indicating the possible involvement of HMGA1 in the Wnt/beta-catenin signalling pathway. Similar results were obtained after HMGA1 depletion in another basal-like breast cancer cell line, MDA-MB-157 (Supplementary Fig. S2).

Overall, these results clearly demonstrate that HMGA1 depletion reverses the transformed phenotype of breast cancer cells, indicating potential regulation of the mesenchymal to epithelial transition (MET) by HMGA1.

### HMGA1 silencing decreases self-renewal

A link between the EMT and the cancer stem cell phenotype has been demonstrated [7]. Thus, given our observations that HMGA1 is involved in the MET, we asked whether HMGA1 influences the cancer stem cell population and its self-renewal capacity. We used a mammosphere-formation assay, in which cancer stem cells can form mammospheres under non-adherent conditions [25]. We found that HMGA1 depletion led to a strong reduction in mammosphere formation and dimension (Fig. 2F). Moreover, to assess whether HMGA1 controls mammosphere self-renewal, we dissociated the primary mammospheres into single cells and reseeded them to evaluate their ability to form secondary mammospheres. HMGA1 depletion maintained mammosphere inhibition in the subsequent passages (Fig. 2G), demonstrating that HMGA1 decreases the self-renewal capacity of mammosphere-forming cells. These data suggest that HMGA1 is involved in the growth and self-renewal capacity of breast cancer stem cells.

### HMGA1 knockdown reduces the malignant features of human breast cancer cells and inhibits their migration and invasion

**Figure 3:**
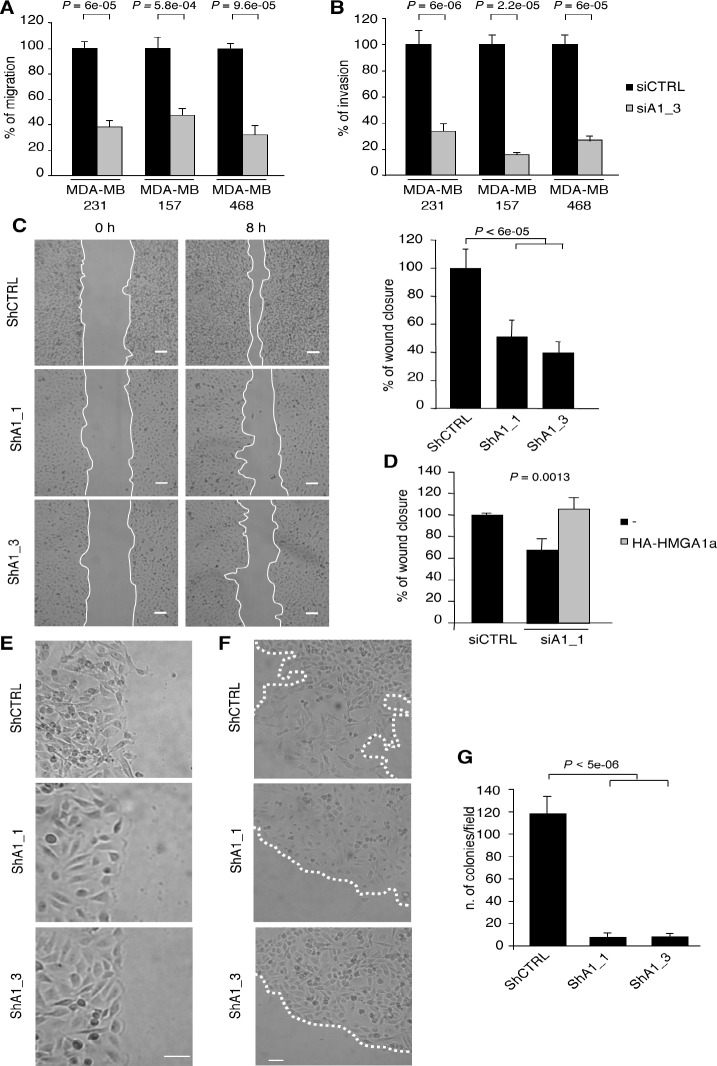
HMGA1 Depletion Inhibits Transformation Characteristics in Breast Cancer Cells (A) Transwell migration assays in MDA-MB-231, MDA-MB-157 and MDA-MB-468 cells transfected with control (siCTRL) and HMGA1 (siA1_3) siRNA. (B) Transwell invasion assays in the cells shown in (A). The data in (A) and (B) are represented as the means of the percentage of the number of cells relative to control ± SD (n=3). See Supplementary Fig. S3A, for the western blot analysis of the cell lysates. (C) Representative pictures from the scratch wound-healing assay, performed with HMGA1-depleted (ShA1_1 and ShA1_3) and control (shCTRL) cells. The scale bar represents 100 μm. Quantification of the scratch wound-healing assay is shown. The data are represented as the means of the percentage of wound closure at 8 hours relative to control ± SD (n=3). (D) The scratch wound-healing assay in MDA-MB-231 cells that have been cotransfected with control (siCTRL) or HMGA1 (siA1_1) siRNA and vectors expressing siRNA-resistant HA-HMGA1a. The empty vector (-) was used as a negative control. The data are represented as the means of the percentage of wound closure relative to control ± SD (n=3). See Supplementary Fig. S3B, for the western blot analysis of the cell lysates. (E) Representative pictures illustrating that the MDA-MB-231 cells in which HMGA1 (ShA1_1 and ShA1_3) was depleted move as a coherent group. The scale bar represents 50 μm. (F) Representative pictures of the cell dispersion assay. The scale bar represents 50 μm. (G) Quantification of anchorage-independent growth in soft-agar. The data are represented as the means ± SD (n=3).

Given that the EMT and CSCs play critical roles in tumour metastasis [7, 26, 27], we next evaluated the impact of HMGA1 depletion on cell motility and invasiveness *in vitro*. HMGA1 depletion significantly reduced both trans-well migration and invasion in MDA-MB-231 cells by at least 60% compared with controls, a result that was later confirmed in two independent basal-like cell lines (MDA-MB-157 and MDA-MB-468) (Fig. 3A and B). Moreover, we confirmed the inhibitory effect of HMGA1 knockdown on migration in wound healing-induced migration assay (Fig. 3C) and demonstrated that this effect was strictly dependent on HMGA1 because the reintroduction of a siRNA-resistant HMGA1 construct almost entirely rescued cell migration (Fig. 3D). During the MET, decreased metastatic ability is deeply connected to the reacquisition of a polarised epithelial phenotype in which cell-cell contacts are restored and the cells move collectively [28]. In fact, we observed that HMGA1-silenced cells migrate collectively to the wound centre, a finding that is consistent with the acquisition of epithelial cell properties, whereas control cells move individually (Fig. 3E). Moreover, using a cell dispersion assay to simulate in 2D the metastatic dispersion of cells from the primary tumour, we observed that HMGA1-depleted cells grow in a monolayer as well-defined colonies, while control cells move individually, breaking the colony boundaries (Fig. 3F). These findings emphasise that HMGA1 promotes the migration and invasion of breast cancer cells by establishing the mesenchymal transition programme.

Both cell survival outside of the primary tumour and metastatic potential depend on the process of anchorage-independent growth, which is a key aspect of the tumour phenotype. Through *in vitro* soft agar assay analysis we observed that inhibition of HMGA1 expression dramatically suppressed the ability of MDA-MB-231 cells to form colonies in soft agar (Fig. 3G), demonstrating that HMGA1 is required for the survival and proliferation of breast cancer cells in the absence of external stimuli. Taken together, these results suggest the functional involvement of HMGA1 in metastatic processes.

### HMGA1 is critical for *in vivo* metastatic processes

**Figure 4:**
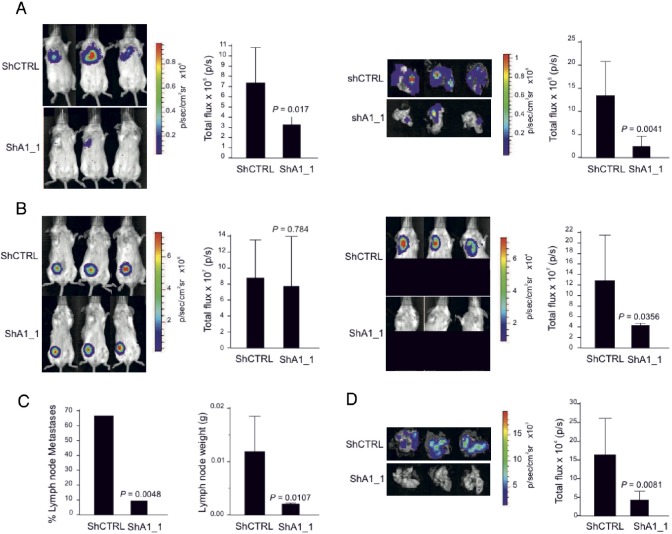
HMGA1 Depletion Suppresses Metastasis in a Mouse Xenograft Model (A) MDA-MB-231 ShCTRL and ShA1_1 cells carrying the firefly luciferase reporter gene were injected into the tail veins and mice were analysed for metastasis using bioluminescence. Three representative mice from each group exhibiting metastasis are shown in the left panel with the corresponding averages of the total flux analyses of 12 mice for each group. Representative *ex vivo* images of the excised lungs and corresponding averages of the total flux analyses are shown on the right. Analyses were performed at day 26 after cell injection. The data are represented as the means ± SD (n=12 for each group of mice). (B) MDA-MB-231 ShCTRL and ShA1_1 cells carrying the firefly luciferase reporter gene were injected into the fat pad. Three representative mice showing primary tumours detected *in vivo* and the corresponding averages of the total flux analyses are shown on the left. Shown on the right are the metastases detected *in vivo* and the corresponding averages of the total flux analyses. Here, the lower portion of each animal was shielded before reimaging to minimise the bioluminescent signal from the primary tumour. (C) The number of metastatic lymph nodes (left) and the average weights of the lymph nodes (right) excised from mice injected with cells in the fat pads. The lymph node weight data are represented as the means ± SD (n=21 for ShCTRL mice and n=21 for ShA1_1 mice). (D) Representative *ex vivo* images of the lungs excised from mice that had been injected with cells in the fat pad and the corresponding averages of the total flux analyses are shown. For (B) and (D), the analyses were performed at day 40 after cell injection. The data are represented as the means ± SD (n=19 for ShCTRL mice and n=21 for ShA1_1 mice).

We next examined the impact of HMGA1 on tumour growth and metastatic dissemination *in vivo*. MDA-MB-231 control cells and HMGA1-depleted cells were genetically engineered to express the firefly luciferase reporter gene, enabling *in vivo* bioluminescence imaging. To evaluate metastatic potential *in vivo*, we conducted a tail vein xenograft in mice. We observed strong reductions in the BLI signal in the lung regions of animals that had been injected with HMGA1-depleted cells (Fig. 4A, left). These data were confirmed via bioluminescence analysis of explanted lungs (Fig. 4A, right). These preliminary *in vivo* results provide evidence of the active regulation of metastatic processes in breast cancer by HMGA1. However, the intravenous injection of tumour cells bypasses several critical steps of the metastatic cascade, including invasion of the tumour border and intravasation into the vasculature. Therefore, we tested whether HMGA1 knockdown impaired metastasis in a more physiologically relevant experimental model by injecting the cells subcutaneously into the fat pads of SCID mice. The results indicated that HMGA1 depletion did not significantly affect tumour growth (Fig. 4B, left). Nevertheless, mice injected with HMGA1-knockdown cells displayed significant reductions in the BLI signal in the region associated with tumour colonisation of the regional homolateral axillary lymph nodes (Fig. 4B, right), which are the primary site of metastatic dissemination [29]. Notably, we found that 14 of 21 control mice displayed lymph node metastasis at this site, whereas only 2 of 21 mice carrying HMGA1-depleted tumours were macroscopically positive (Fig. 4C, left). Accordingly, a highly significant reduction in lymph node weight was also observed (Fig. 4C, right). Finally, this observation was further confirmed by an *ex vivo* BLI analysis of the lungs (Fig. 4D), which revealed dramatic reductions in lung colonisation from the naturally occurring MDA-MB-231 metastases. Hence, the results of the experiments outlined above indicate that HMGA1 may control both migratory and invasive behaviours in breast cancer cells *in vivo*.

### The HMGA1 gene signature is an independent predictor of poor clinical outcome

To understand the functional involvement of HMGA1 in breast cancer malignancy we investigated whether HMGA1 alters the transcriptional programme by analysing the transcriptional profile of MDA-MB-231 cells in the presence and absence of HMGA1. Following HMGA1 silencing, we identified the gene cluster containing the genes that were most up-regulated and a larger cluster containing the genes that were most down-regulated (siHMGA1UP genes, n=38 and siHMGA1DW genes, n=130 genes respectively, see also Supplementary Fig. S4 and Table S1 and S2), consistent with notion that HMGA1 acts predominantly to activate transcription. The effects of HMGA1 depletion on the expression of selected genes were confirmed by qRT-PCR (Supplementary Fig. S5). A functional annotation analysis of the HMGA1 transcriptome, performed with the DAVID/EASE and Ingenuity Pathway Analysis (IPA) tools, led to the conclusion that HMGA1 silencing affects genes involved in cancer and regulation of the cell cycle, cellular movement, growth, proliferation and metabolism (Table 1 and Supplementary Table 3).

**Table 1:** Functional annotation of HMGA1 modulated-genes

DOWN regulated genes upon siHMGA1		UP regulated genes upon siHMGA1	
Functional Analysis		Functional Analysis	
DAVID	p-value	DAVID	p-value
cell cycle	8.49E-12	regulation of kinase activity	0.031
M phase	6.40E-09	domain:Cadherin 7	0.034
condensed chromosome	1.57E-06	regulation of transferase activity	0.034
nuclear lumen	2.10E-06	FAD-dependent pyridine nucleotidedisulphide	0.038
		oxidoreductase	
nuclear division	1.23E-05	EGF	0.043
mitosis	1.23E-05		
**Ingenuity Pathway Analysis**	**p-value**	**Ingenuity Pathway Analysis**	**p-value**
Cancer	4.56E-08 - 3.84E-02	Cancer	3.92E-06 - 1.73E-02
Reproductive System Disease	2.25E-04 - 3.84E-02	Gastrointestinal Disease	3.92E-06 - 1.73E-02
Cell Cycle	3.48E-13 - 3.68E-02	Cellular Movement	3.32E-05 - 1.73E-02
DNA replication and Repair	1.45E-08 - 3.30E-02	Cellular Growth and Proliferation	4.88E-05 - 1.73E-02
Embryonic Development	2.25E-05 - 3.84E-02	Lipid Metabolism	1.71E-04 - 1.73E-02
Hematological System Development	3.04E-04 - 3.30E-02	Cell-Mediated Immune Response	3.32E-05 - 1.16E-02

Starting from the genes that were expressed in the silenced HMGA1 cells that had greater than a 1.4 log-fold change or lower than a 1.4 log-fold change with respect to the control cells, we used the publicly accessible software DAVID/EASE and Ingenuity Pathway Analysis. These are tools that can be used to summarise the predominant biological “theme” of a given gene list with respect to all of the genes represented in the dataset. The most over-represented terms (p<10^−5^) in the down-regulated gene cluster in the silenced HMGA1 cells were related to the mitotic cell cycle and mitosis (upper panel), while the up-regulated gene cluster was characterised by GO and was related to metabolism, movement and proliferation (lower panel). The complete GO term list is available in the supplementary files (See Supplementary Table S3).

**Figure 5:**
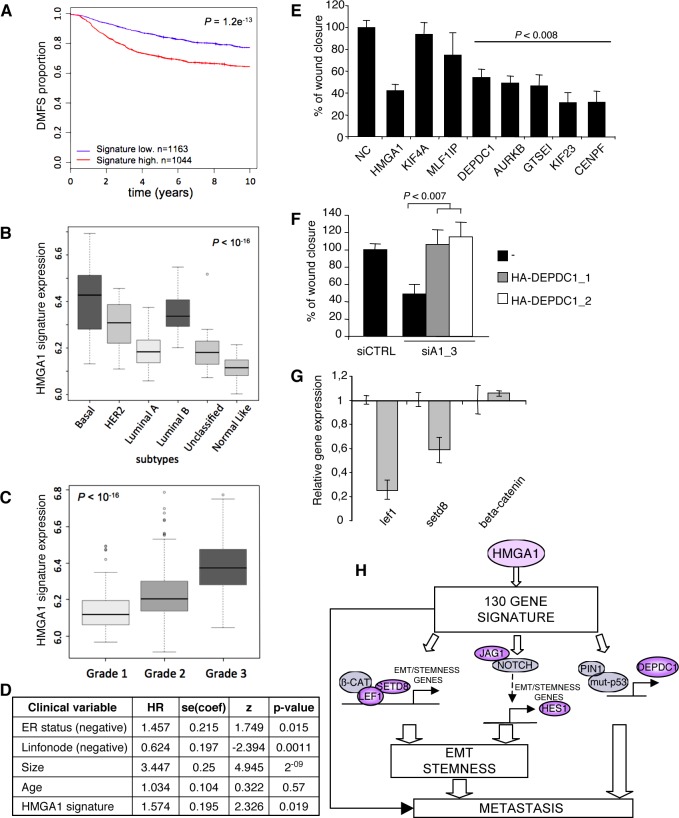
HMGA1 Gene Signature and Breast Cancer Phenotype (A) Kaplan–Meier survival curve of time to distant metastasis (TDM) for breast cancer patients who were classified according to HMGA1 expression. Red line: cases with high HMGA1 expression; blue line: cases with low HMGA1 expression. (B-C) Boxplots of the distribution of the gene expression intensities of the HMGA1 gene signature across different breast cancer subtypes (intrinsic subtypes or Grades 1, 2 or 3). (D) Multivariate analysis of risk of death. (E) The scratch wound-healing assay in MDA-MB-231 cells transfected with control siRNA (negative control, NC) or siRNA specific for each analysed gene. The data are represented as the means of the percentage of wound closure relative to control ± SD (n=4). (F) The scratch wound-healing assay in MDA-MB-231 cells that have been cotransfected with control (siCTRL) or HMGA1 (siA1_3) siRNA and vectors expressing two DEPDC1 isoforms (DEPDC1-1 and DEPDC1-2). The empty vector (-) was used as a negative control. The data are represented as the means of the percentage of wound closure relative to control ± SD (n=3). See also Supplementary Fig. S7, for the western blot analysis of the cell lysates. (G) Down-regulation of Wnt-related genes after HMGA1 silencing (grey bar) was measured using real-time PCR. Expression was normalised to the levels in MDA-MB-231 cells transfected with control siRNA. GAPDH was used as an internal control. The data are represented as the means ± SD (n=3). (H) Proposed model for the role of HMGA1 in basal-like breast cancer. HMGA1 regulates a panel of 130 genes that are critical for migration, EMT and stemness and that strongly impact breast cancer. Among these genes, LEF1 and SETD8 are key factors in the Wnt/beta catenin pathway, JAG1 and HES1 are key factors in the Notch pathway, and DEPDC1 is a key factor in the mutant p53 pathway.

To address the hypothesis that the transcriptional programme induced by HMGA1 contained genes that are important for tumour aggressiveness, we initially utilised the Oncomine web tool [30, 31]. Interestingly, the results of this analysis clearly revealed higher expression of the genes that were down-regulated after HMGA1 silencing (i.e., genes induced by HMGA1) in tumour tissue vs. normal tissue (ratio 89/6), and in particular in bad vs. good clinical outcome (ratio 55/4), primarily for breast cancer (ratio 31/0) (Table 2). Therefore, to further evaluate this clinical correlation, we analysed several breast cancer microarray datasets, which collectively consisted of more than 2000 patients (breast cancer meta-dataset). A Kaplan-Meier survival analysis showed that the expression of these genes was significantly correlated with clinical outcome. In particular, patients expressing high levels of these genes displayed a shorter time to distant metastasis (TDM) (Fig. 5A). In addition, the higher HMGA1 expression gene signature was associated with both the basal-like subtypes and high-grade (G3) breast cancers (Fig. 5 B and C) and was also correlated with HMGA1 mRNA expression (Supplementary Fig. S6).

**Table 2:** Oncomine analysis of HMGA1 regulated-genes

Cancer Type	Cancer vs. Normal		Clinical Outcome	
	Over-expression or Copy Gain	Under-expression or Copy Loss	Over-expression or Copy Gain	Under-expression or Copy Loss
Bladder Cancer	2		1	
Brain and CNS Cancer	5	1	4	
Breast Cancer	8	1	31	
Cervical Cancer	2			
Colorectal Cancer	14		2	1
Esophageal Cancer	3			
Gastric Cancer	4			
Head and Neck Cancer	10			
Kidney Cancer			2	
Leukemia	1	3		
Liver cancer	4			
Lung Cancer	13		4	
Lymphoma			3	3
Melanoma	1			
Myeloma			5	
Other Cancer	5	1	2	
Ovarian Cancer	4			
Pancreatic Cancer	2			
Prostate Cancer	2		1	
Sarcoma	10			
**Significant Unique Concepts**	**89**	**6**	**55**	**4**
**Ratio**	**14.83 (p<0.0007)**		**13.75 (p<0.009)**	

Starting with the genes expressed in the silenced HMGA1 cells that displayed less than a 1.4 log-fold change with respect to the control cells (HMGA1 signature), we used the Oncomine (PRO version) web tool to determine if there were any associations between the gene expression profiles of the different cancer types present in the database. This table displays the number of significant results, coloured red or blue for over- or under-expression, respectively, across all cancer types, with an analysis of the correlation with clinical outcomes. P-values were calculated using a two-sample paired Wilcoxon test (also known as the ‘Mann-Whitney’ test).

Next, we assessed whether this signature could be an independent predictor of clinical outcome. Cox multivariate analysis revealed that the HMGA1 gene signature behaves as a significant (p<0.05) independent prognostic factor together with ER status, lymph node impairment and tumour size (Fig. 5D). In addition, after analysing a cohort of 115 patients that had more clinical variables available, we confirmed that the HMGA1 signature yields prognostic information when combined with clinical variables that are commonly used in practice (Supplementary Table S4). Hence, the genes up-regulated by the HMGA1 protein have prognostic significance, and their combined expression may be considered as a marker of breast cancer malignancy. Taken together, these data suggest that HMGA1 plays a key role in breast malignancy and the progression of metastatic disease by modulating a specific gene network.

### HMGA1 activates aggressiveness-related and stemness-associated factors

Given our observations that HMGA1 increases migration and invasion and supports EMT and breast cancer stemness, we assessed the functional roles of the HMGA1 gene signature. First, we selected seven of the most differentially expressed genes that were correlated with clinical data outcomes (i.e., GTSEI, AURKB, CENPF, MLF1IP, DEPDC1, KIF23 and KIF4A) and evaluated their involvement in the HMGA1-mediated reduction of migration ability. These genes are known to be strongly associated with the microtubule network and involved in cytoskeletal organisation [32-36]. To establish their role in cell motility, we explored the effects of silencing these genes in MDA-MB-231 cells using a wound-healing assay. We observed that the depletion of five of the seven genes (GTSEI, AURKB, CENPF, DEPDC1 and KIF23) significantly inhibited cell migration (Fig. 5E). Among these five genes, emerging evidence suggests that DEPDC1 plays an important role in bladder cancer and breast cancer cells [37, 38]. In particular, DEPDC1 has been reported to have a strong impact on migration and invasiveness that is dependent on mutant p53 [37]. To further evaluate whether DEPDC1 acts as an effector in HMGA1-induced breast cell transformation, we transiently transfected DEPDC1 into MDA-MB-231 cells in which HMGA1 expression had been silenced. We observed that DEPDC1 overexpression rescued the effects of HMGA1 silencing on migration (Fig. 5F). Therefore, our results suggest that HMGA1 plays a pivotal role in orchestrating a molecular network that sustains the invasiveness of cancer cells.

Among the genes regulated by HMGA1, we found different genes known to be involved in processes related to the EMT and the formation of stem cells, including CD24, FAM83H, IL1R1, SERPINE1, CALD1, TUBB, LIFR, LEF1 and SET8. Interestingly, LEF1 and SET8 are regulatory elements of the Wnt/beta-catenin pathway that cooperate in a complex with beta-catenin and function as coactivators to sustain the EMT and stem properties [39]. We confirmed the down-regulation of LEF1 and SET8 mRNA using qRT-PCR. Beta-catenin mRNA levels did not change (Fig. 5G). However, we observed protein relocalisation (Fig. 2D), indicating that HMGA1 cooperates with the Wnt/beta-catenin signalling pathway to sustain the EMT and stemness.

## DISCUSSION

This study aimed to deepen our understanding of the role of HMGA1 in breast cancer metastasis.

We demonstrated that HMGA1 plays a pivotal role in regulating invasive processes and determining poor prognostic outcomes in breast cancer by sustaining the mesenchymal phenotype and stemness. In fact, silencing HMGA1 in highly metastatic breast cancer cell lines impaired mesenchymal fibroblastoid features. Cells undergo a phenotypic reversion, reacquiring a more differentiated, polarised epithelial phenotype, which is attributable to EMT regression. During the EMT, cells lose cell-cell contacts and gain an increased ability to migrate from the primary tumour and invade surrounding tissues [24]. We clearly demonstrated that breast cancer cell lines in which HMGA1 has been depleted behave as an organised epithelial sheet and that their migration and invasion ability are dramatically decreased both *in vitro* and *in vivo*, even if tumour growth is not impaired. Over the past few years, close crosstalk between the EMT and stemness has emerged, which explains most of the steps in the invasion-metastasis cascade [7, 8]. Using bioinformatic analyses, we demonstrated that HMGA1 is overexpressed in the breast cancer subtypes that carry poor prognoses and tend to metastasise. Moreover, we demonstrated that breast cancer cells in which HMGA1 has been depleted lack self-renewal capability and lose the ability to form mammospheres in culture. Cancer stem cells are considered to be prime candidates for the initiation of relapse and the most important features of macrometastasis formation [6]. The results of the present study suggest a novel mechanism by which HMGA1 controls the progression of tumour cells to an aggressive and invasive phenotype: aberrant expression of HMGA1 in tumour cells promotes metastasis by inducing EMT- and stemness-related processes, which in turn may enhance the ability of breast cancer cells to migrate and grow at secondary sites.

Our study may also have clinically relevant predictive implications. Several reports have provided strong evidence that breast cancer prognosis can be derived from the gene expression profile of the primary tumour. Notably, we found an HMGA1 gene-signature that significantly overlaps with different gene signatures that identify patients with poor prognosis and a high risk of distant metastases. Indeed, some of the genes in the HMGA1 signature (CENPF, CENPA, CCNE2, BUB1 and PSMD2) are also part of the 70-gene prognosis profile [40]. In line with our results relative to HMGA1 expression in primary tumour specimens from breast cancer patients, we demonstrated that the HMGA1 gene signature is correlated with the more aggressive and undifferentiated basal-like subtype, with high relapse rates and poor patient survival. Moreover, these gene signatures could potentially be used to select a group of patients at a specific state of the disease, facilitating the selection of treatments [40, 41]. Intriguingly, our data indicate that the HMGA1 gene signature is a strong independent factor in the prediction of disease outcome. In particular, it is associated with poorer clinical outcomes that are related to a short time to distant metastasis.

Our microarray analyses indicate that HMGA1 may directly regulate the expression of a number of genes that are biomarkers for prognosis, relapse and metastasis (Fig. 5H). Consistent with this conclusion, we demonstrated that 5 of the 7 genes tested were directly involved in promoting breast cancer cell migration. These genes are implicated in microtubule dynamics and, among them, GTSE1 has emerged as a microtubule-associated protein that is correlated with tumour metastasis in breast cancer. GTSE1 promotes migration via focal adhesion turnover [42], suggesting a possible role for HMGA1 in controlling migration through the regulation of microtubule pathways. In the present study, we found a significant overlap of our gene signature with the recently defined Pin1/mutant p53 gene signature [37, 43]. In agreement with previous findings indicating that DEPDC1, a direct downstream target of the mutant p53 pathway, is relevant to migration and invasion, we found a direct link between HMGA1 and DEPDC1. Both genes regulate migration, suggesting that HMGA1 may cooperate with the mutant p53 pathway to modulate breast cancer aggressiveness. Furthermore, using the Molecular Signatures Database (MSigDB), we found significant overlap between the HMGA1 signature and the genes regulated by YB-1, a multitasking member of the cold-shock domain protein superfamily. YB-1 activates genes encoding EMT-associated proteins and promotes stem cell properties in breast cancer [44, 45]. The overlap between the YB-1 and HMGA1 gene signatures further illustrates the fact that HMGA1 is involved in breast cancer aggressiveness that is mediated by EMT and stem cell processes.

The results of the present study provide evidence that HMGA1 regulates the crucial processes of the EMT, stemness and sustained metastasis. One of the critical pathways that regulate stemness and the acquisition of EMT characteristics during tumourigenesis and metastasis is the Wnt/beta-catenin pathway [46, 47]. In particular, beta-catenin translocates from adhesion junctions to the nucleus to regulate genes that are involved in promoting stemness and the EMT. Surprisingly, HMGA1 depletion induces a dramatic relocalisation of beta-catenin to cell-cell contact points and likely modulates the expression of two beta-catenin coactivators, Lef1 and Setd8, that interact directly as mediators of Wnt signalling [39]. The Wnt and Notch pathways work together during carcinogenesis to induce self-renewal [47]. Moreover, the Notch pathway regulates the EMT in both physiological and pathological conditions [26]. Interestingly, HMGA1 silencing down-regulates Jag1 and its downstream effector Hes1, allowing crosstalk to occur between HMGA1 and the Wnt and Notch pathways. These findings collocate HMGA1 as a critical hub in the regulation of relevant pathways that promote the EMT and stemness in breast cancer.

The results of our study not only suggest that HMGA1 plays a critical role in breast cancer aggressiveness but also provide strong evidence for the action of HMGA1 in basal-like breast tumours. Therefore, the biological insights generated by this study may assist in the development of new therapeutic strategies for basal-like breast cancer subtypes that are associated with poor prognoses.

## MATERIAL AND METHODS

### Cell Culture and Transfection

MDA-MB-231 and MDA-MB-157 cells were grown in DMEM plus 10% tetracycline-free FBS, MDA-MB-468 cells in RPMI 1640 plus 10% tetracycline-free FBS. For transfection of siRNAs, all cell lines were transfected with 100 nM siRNAs with Lipofectamine™ RNAiMAX reagent (Invitrogen). For plasmid, transfection was performed with FuGENE (Roche). For the functional-rescue experiment, cells were first transfected with siRNAs and with plasmid 24 hours later.

### Design of Inducible ShRNA Cell Lines

MDA-MB-231 cells were transfected with the vector pcDNA6/TR™ (Invitrogen) Cells were selected for the presence of the plasmid in appropriate medium with 5 μg/ml Blasticidin (Sigma), and the surviving clones were chosen and amplified. A clone with a high expression of the Tet-repressor (called MDA-MB-231 Tet-R) was chosen. Three shRNAs were designed as previously described [48]; shCTRL was used as a negative control, and shA1_1 and shA1_3 were designed against 3'UTR and ORF of HMGA1, respectively. The oligonucleotides were cloned into the doxycycline-inducible vector pSUPERIOR.neo (OligoEngine). MDA-MB-231 Tet-R cells were transfected with these vectors and selected in the appropriate medium with 1 mg/ml G-418 (Sigma). The surviving clones were chosen and amplified. Two clones for each short hairpin, namely ShCTRL, ShA1_1 and ShA1_3 were selected, and all of the assays were performed in 1 ug/ml doxycycline (Sigma) to induce shRNA expression.

### Growth in 3D

Cells were seeded at 20,000 cells per well in 24-well plates precoated with Laminin-rich extracellular matrix, Matrigel, (BD Biosciences) as previously described [23]. Cells were grown for 11 days. The colonies were photographed with a digital Canon PowerShot A630 camera.

### Mammosphere culture

Mammosphere Assay was performed as previously described with modifications^25^. 5000 cells/well were seeded in 6-well ultra-low adhesion plates (Corning) in MEGM medium, containing 2% methylcellulose supplemented with 20 ng/ml EGF (Stem Cell Technologies), 10 ng/ml bFGF (Orfgenetics), and B27 (GIBCO). For secondary and tertiary sphere formation, primary and secondary spheres were dissociated by trypsinization and plated at 5000 cells/well.

### Migration and Invasion Assays

For wound healing assays, cells were cultured to 90% confluence on 35-mm plates. The cells were then scraped with a 200-μl tip, and wound closure was followed for 8 hours. Images of same area were taken for each plate. For transwell migration and invasion assays, 24-well PET inserts were used (8.0 mm pore size, Falcon) with matrigel-coated filters for invasion and 100,000 cells were seeded. Migrated cells were fixed in PFA 4% and stained with Crystal Violet 0.5% (Sigma).

### Cell Proliferation

Cells were seeded in 6-well plates to a final density of 100,000 cells per well. Proliferation was examined every 24 hours by cell counts.

### Growth in Semisolid Medium

The bottom layer was obtained by covering 6-well dishes with 3 ml of 0.6% agar in DMEM. 10,000 cells were seeded on top in 2 ml of 0.3% agar in DMEM. Colonies were grown for 3 weeks, then stained with 0.5 mg/ml MTT (Sigma) and counted.

### Protein Extraction and Western Blot Analysis

Cells were washed in chilled PBS and lysed using SDS sample buffer. Western blot analyses were performed according to standard procedures.

### Immunostaining

Cells were grown on glass slide and fixed with PFA 4%. After permeabilization with 0.3% Triton/PBS and saturation in 0.5% BSA/PBS, cells were incubated for 1 hour at RT with primary antibodies diluted in 0.5% BSA/PBS. Secondary antibodies were applied for 1 hr at RT, and the cells then were stained with Hoechst. For F-Actin staining, Phalloidin-conjugated Alexa Fluor 488 (Invitrogen) was used for 1 hour at RT at a concentration of 1:40 in PBS. The images were visualized by a Nikon Eclipse e800 microscope and acquired by Nikon ACT-1 software.

### Gene Expression Analysis

Total RNA was extracted using Trizol reagent (Invitrogen) subjected to DNase-I (Invitrogen) treatment and subsequently column-purified with RNeasy kits (QIAGEN). For microarray analysis, four biological mRNA replicates for each group (siCTRL or siA1_3) were hybridized on Affymetrix GeneChip Human Genome U133A 2.0 array. For quantitative RT-PCR, mRNA was transcribed using Superscript II (Invitrogen). Quantitative PCR was performed using SYBR Green PCR master mix (Applied Biosystems) and 7500 Real-Time PCR System (Applied Biosystems).

### Cellular Transduction

For *in vivo* metastasis assays, MDA-MB-231 cells were co-transduced with a lentiviral vector coding for the Firefly Luciferase reporter gene. The vector was produced in 293T cells by transient co-transfection of the transfer (pHR'tripCMV-luc2-IRES-tNGFR-SIN), envelope (hCMV-G) and packaging plasmids (p8.74) as previously described [49]. Cells were cultured and expanded.

### Mouse Strain and Animal Care

We used SCID female mice (Charles River Laboratories, Lecco, Italy) aged 7 weeks for *in vivo* studies. Mice were administered drinking water supplemented with 4% sucrose plus 2 mg/ml Doxycycline to induce shRNA expression (shCTRL and shA1_1). Doxycycline-supplemented water was changed every 2 days. Procedures involving animals and their care were in conformity with the institutional guidelines (D.L. 116/92 and subsequent complementing circulars), and all experimental protocols were approved by the Ethical Committee of the University of Padua (CEASA).

### *In vivo* Experiments

For the intravenous injection, 100,000 cells were resuspended in 200 μl of DMEM for each mouse, whereas for the fat pad injection, 1 million of cells were resuspended in 100 μl of DMEM. We performed *in vivo* imaging at 20 and 26 days after i.v. injection or at 14, 21, 28, 34 and 40 days after fat pad injection. Anesthetized animals (1-3% isoflurane, Merial Italia S.p.A, Italy) were given the substrate D-Luciferin (Biosynth AG, Switzerland) by intraperitoneal injection at 150 mg/kg in PBS (Sigma). Imaging times ranged from 15 seconds to 5 minutes, depending on the tumor model and time point. The light emitted from the bioluminescent tumors or metastasis was detected using a cooled charge-coupled device camera mounted on a light-tight specimen box (IVIS Lumina II Imaging System; Caliper Life Sciences, Alameda, CA). Regions of interest from the displayed images were identified around the tumor sites or metastasis regions, such as the lymph node and lungs, and quantified as total photon counts (photon/s) using Living Image® software (Xenogen). In some experiments, the lower portion of each animal was shielded before reimaging to minimize the bioluminescence from the primary tumor to ensure that the signals from the metastatic regions could be observed *in vivo*. For *ex vivo* imaging, 150 mg/kg of D-Luciferin was injected into the mice just before necropsy. The lungs were excised and imaged for 5 minutes.

### Low-Level Analysis

For microarray analysis, three biological mRNA replicates for each group (siHMGA1 or siControl) were hybridized on Affymetrix hgu133plus2 chips. Cell intensity values were computed using the Affymetrix Expression Console. Further data processing was performed in the R Computing Environment version 2.14 (http://www.r-project.org/) with BioConductor packages (http://www.bioconductor.org/). Robust Multi-Array Average (RMA) normalization was applied [50]. Statistical analysis for differentially expressed genes was performed with limma [51]. P-values were adjusted for multiple testing using the Benjamini and Hochberg's method to control the false discovery rate [52]. Genes with adjusted p-values below 10^−4^ and fold change greater than 2.6 (log 1.4) or lower than −2.6 (−log 1.4) were considered differentially expressed. Gene annotation was obtained from R-Bioconductor metadata packages, and the probesets were converted in Entrez Gene Id and Symbol Id.

### Cluster Analysis

Starting from the normalized annotated expression matrix after gene median centering, features that had standard deviation of less than 0.3 were filtered out. Unsupervised hierarchical cluster analysis (average-linkage method) was performed using Cluster software (EisenLab). Cluster results were then visualized using Java TreeView.

### Functional Analysis

Differentially expressed gene lists obtained from low-level procedures were analyzed for functional associations.

- Data were analyzed through DAVID Bioinformatics Resources v6.7 [53, 54] using the suggested standard parameters.
- Data were analyzed through Ingenuity Pathway Analysis (IPA) software. Core analysis was performed, and the top associated networks table was reported.
- Data were analyzed through the Oncomine Pro web tool using suggested standard parameters. Custom concept analysis was performed, and the “Summary view” (adapted) was reported.

### Breast Cancer Data and Survival Analysis

Several published gene expression datasets (breast cancer meta-dataset) were considered and compared with our HMGA1 dataset. The raw data were retrieved from the gene expression omnibus (GEO) public gene expression database (GSE1456, GSE4922, GSE5327, GSE6532, GSE7390, GSE11121, GSE12093, GSE2603, GSE16446, GSE19615, GSE20685, GSE21653). Data were normalized in R/Bioconductor environment using the RMA normalization method (affy package), creating a breast cancer meta-dataset.

Gene annotation was obtained from brainarray custom CDF metadata packages, and the probesets were converted to Entrez Gene Id and Symbol Id.

Each dataset was analyzed separately to avoid platform and signal merging problems, and only the results were combined together.

To evaluate the correspondence between the HMGA1 expression levels and breast cancer clinical data, we utilized the gene expression-based Outcome for Breast Cancer web tool (GOBO) [55]. To verify the correlation of the HMGA1-gene signature and breast cancer clinical data, a Mantel-Haenszel test was applied to the normalized meta-dataset (survival R package), and the Kaplan–Meier survival curve of time to distant metastasis (TDM) of breast cancer patients classified according to the expression of HMGA1 signature was obtained.

With the same meta-dataset, we searched for the distribution of the gene expression intensities of HMGA1 signature across different breast cancer subtypes (stats R package) and the correlation between HMGA1 expression and the HMGA1 signature expression levels.

### Statistical Analysis

Data were analyzed by a two-tailed Student's t test, and results were considered significant at a p-value < 0.05. The results are presented as the mean and standard deviation (±SD).

### ACCESSION NUMBERS

The accession number in the Gene Expression Omnibus public database for the MDA-MB-231 expression array experiment is GSE35525.

## Supplementary file


