# Supplementary material for: HMGA1 promotes metastatic processes in basal-like breast cancer regulating EMT and stemness
Source: Oncotarget. 2013 Jul 9;4(8):1293–308. doi: 10.18632/oncotarget.1136 (PMC3787158; doi:10.18632/oncotarget.1136)
Supplement: Supplementary file 1 [file oncotarget-04-1293-s001.doc]

SUPPLEMENTARY FIGURES


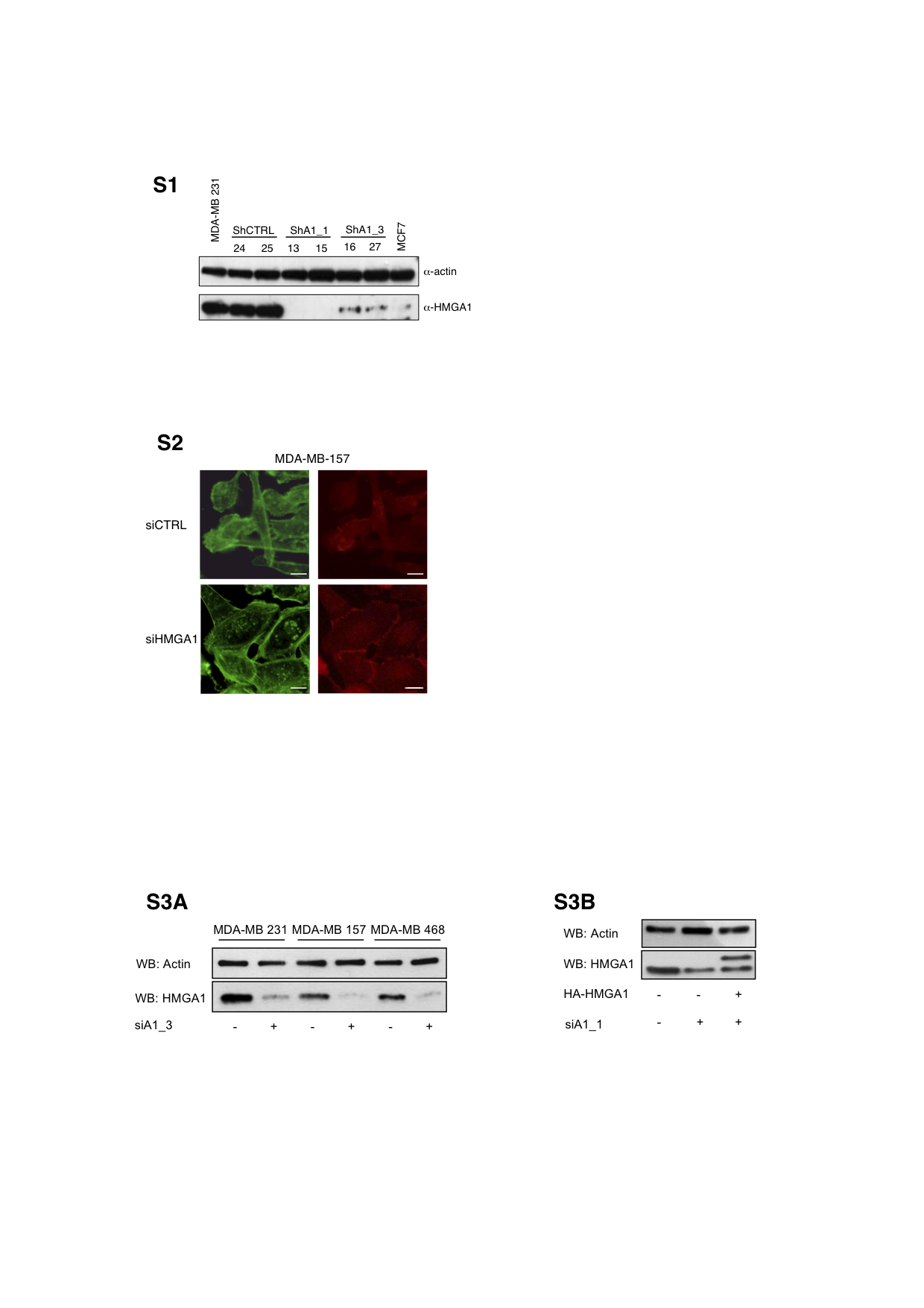


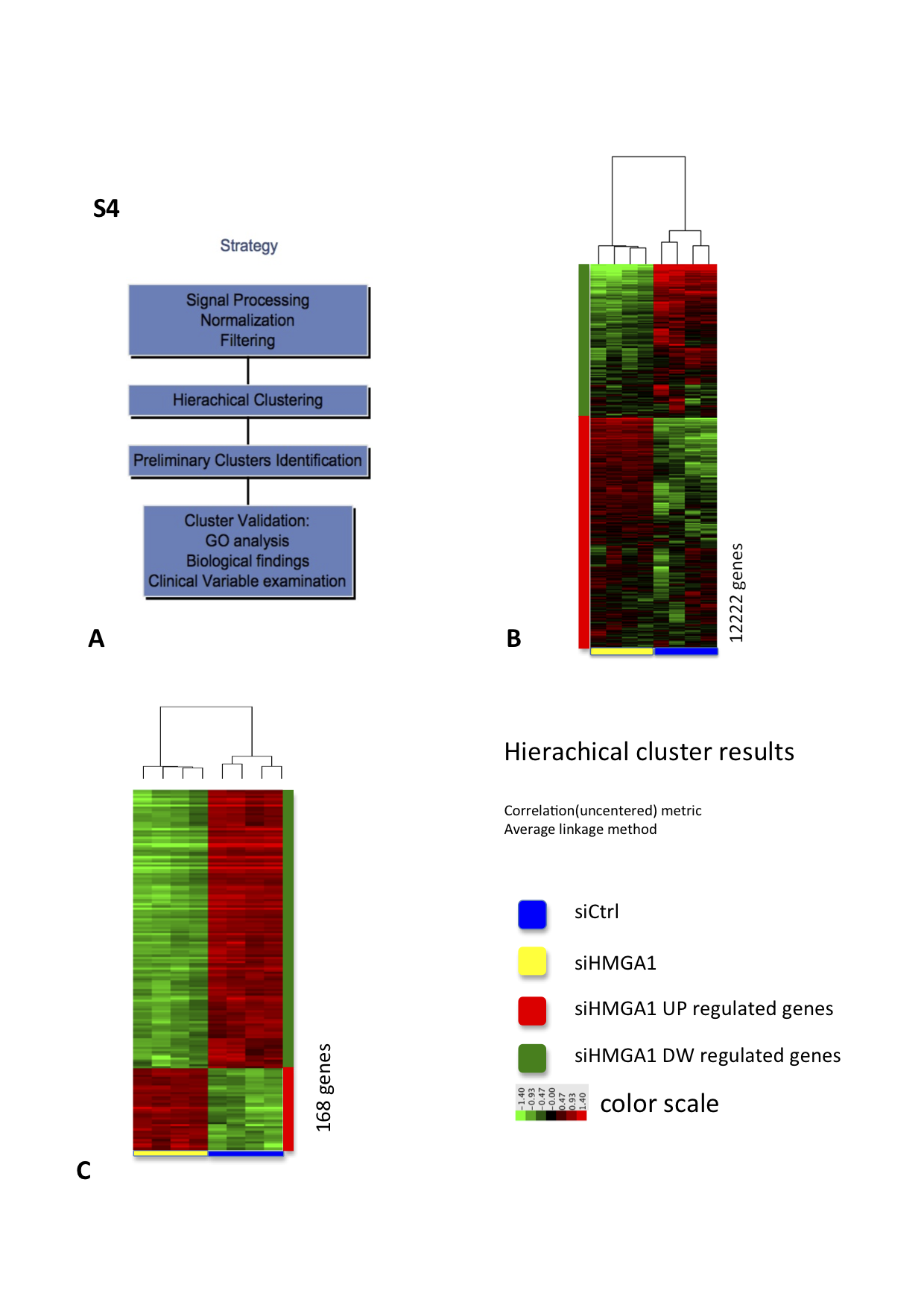


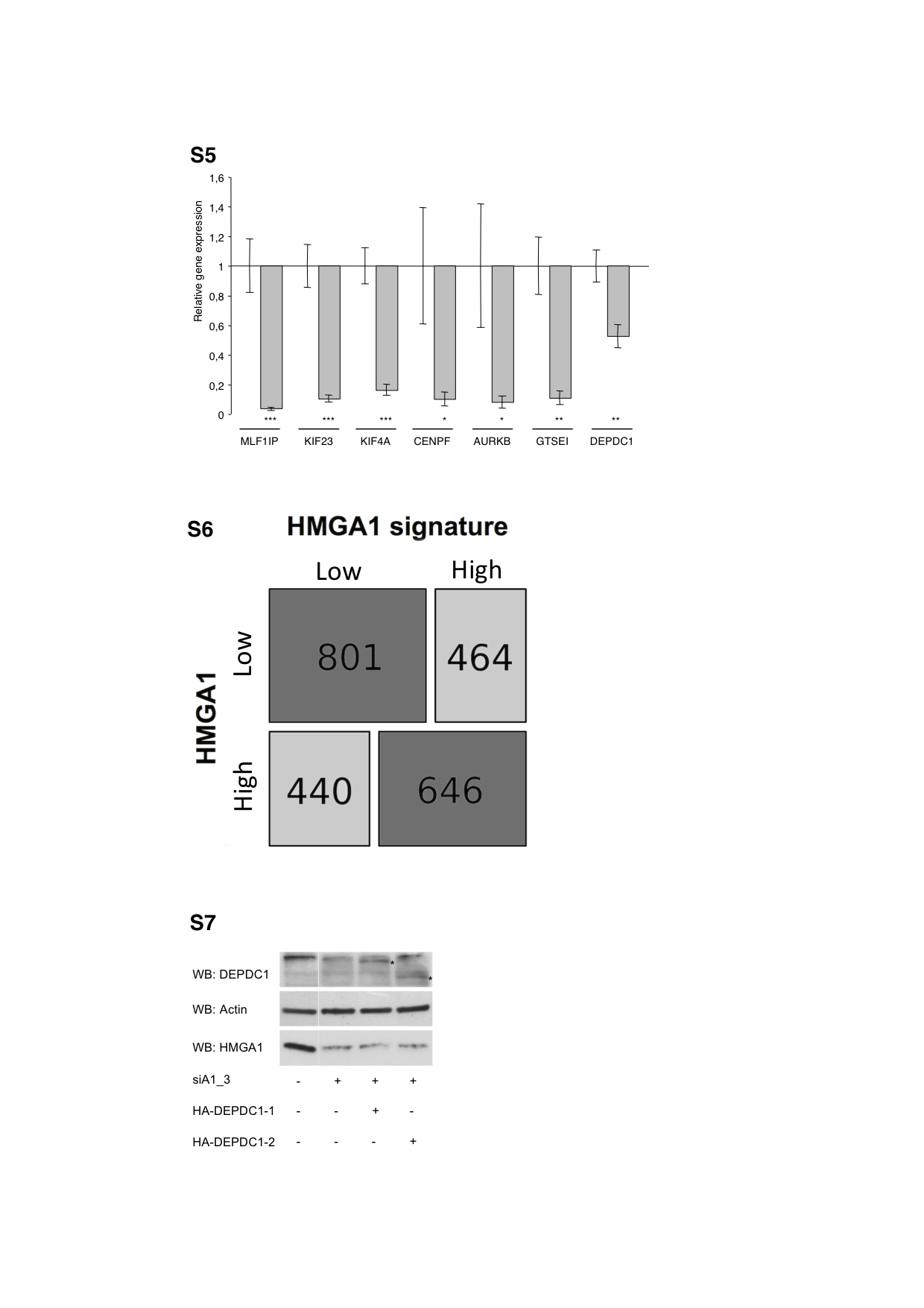


SUPPLEMENTARY FIGURE LEGENDS

Figure S1. HMGA1 depletion in inducible clones after doxyxycline addition. Western blot analysis of HMGA1 levels for different clones of the MDA-MB-231 inducible ShRNA cell lines after doxycycline addition. After induction of the shRNAs, the HMGA1 protein levels were comparable to those of the non-invasive and more differentiated MCF7 breast cancer cell line. The expression of the negative control shRNA (shCTRL) did not affect the HMGA1 protein levels compared with MDA-MB-231 wild-type cells.

Figure S2. HMGA1 depletion in the MDA-MB-157 breast cancer cells. Representative images of the MDA-MB-157 cells transfected with control (siCTRL) or HMGA1 (siHMGA1) siRNA and stained for F-actin (green) and beta-catenin (red). Scale bar represents 10 μm.

Figure S3. Western blot analysis. A, Western blot analysis of the HMGA1 levels after transfection of the MDA-MB-231, MDA-MB-157 and MDA-MB-468 cells with control (-) or HMGA1 (siA1_3) siRNA. Actin was used as an internal control. B, Western blot analysis of the endogenous and transfected (HA-HMGA1) HMGA1 levels in the MDA-MB-231 cells used in the scratch experiment. Actin was used as an internal control.

Figure S4. Microarray analysis of MDA-MB-231 breast cancer cell after HMGA1 depletion. Using the Affymetrix microarray technology, we characterized the gene expression profiles of human MDA-MB-231 cell lines with HMGA1-depleted and control cells. Panel A shows a general schema of our approach, particularly the low level analysis and cluster discovery performed in the R/Bioconductor environment, cluster analysis performed using the Cluster3 (Eisein Lab) tool and cluster annotation performed using the DAVID/EASE analysis web tool. In panel B, the results of the cluster analysis for the global gene expression matrix in which only genes with no variation (standard deviation less than 0.15) were filtered out (tot). Significantly, we can observe the relevance of the impact of HMGA1 reduction on global gene expression. In panel C, the results of the cluster analysis for the genes obtained from differential genes analysis are displayed.

Figure S5. Gene expression analysis after HMGA1 silencing in MDA-MB-231 cells. Downregulation of selected genes after HMGA1 silencing (gray bar) was measured by qRTPCR. Expression was normalized to the level in MDA-MB-231 cells transfected with siCTRL; GAPDH was used as an internal control. Data are presented as the mean ± SD (n=3) (***p<0.001; **p<0.01; *p<0.05).

Figure S6. Correlation between HMGA1 and HMGA1-gene signature expression. We classified all of the breast cancer samples (from the public microarray datasets used in previous analyses) using the expressions of HMGA1 or corresponding signature obtained by our microarray experiments to evaluate their association. The mosaic plot shows the proportion of the four possible groups (High_HMGA1-High_HMGA1_signature, High_HMGA1-Low_HMGA1_signature, Low_HMGA1-High_HMGA1_signature, and High_HMGA1-Low_HMGA1_signature) along with the number of samples in each group. Statistical analysis (using the Pearson's Chi-squared test) showed a significant correlation (p< 2.2e-16).

Figure S7. Western blot analysis of HMGA1 levels and HA-DEPDC1 (see stars) in the MDA-MB-231 cells used in the scratch experiment. Actin was used as an internal control.

SUPPLEMENTARY TABLES

Table S1. Up-regulated genes after HMGA1-silencing


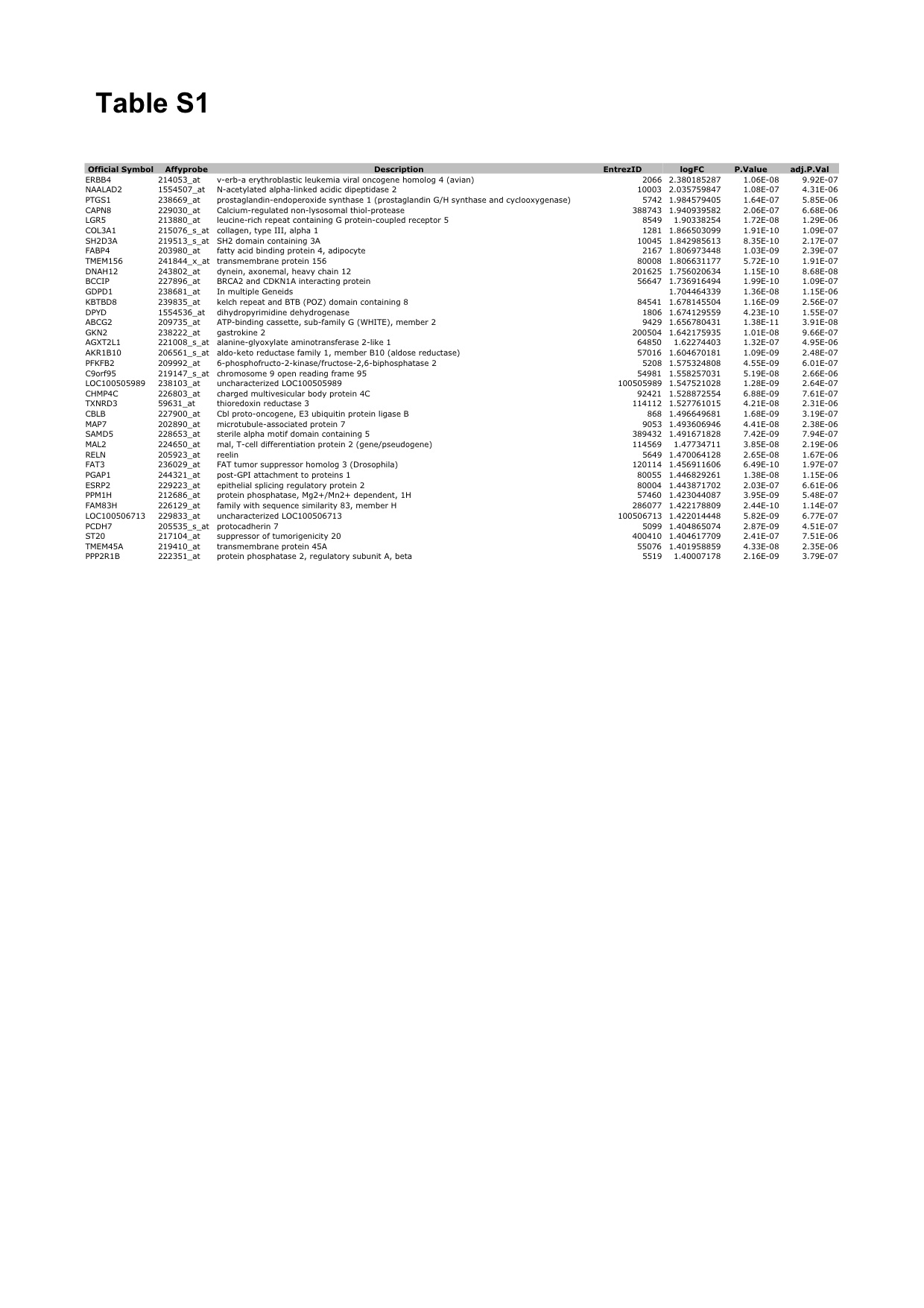


Table S2. Down-regulated genes after HMGA1-silencing


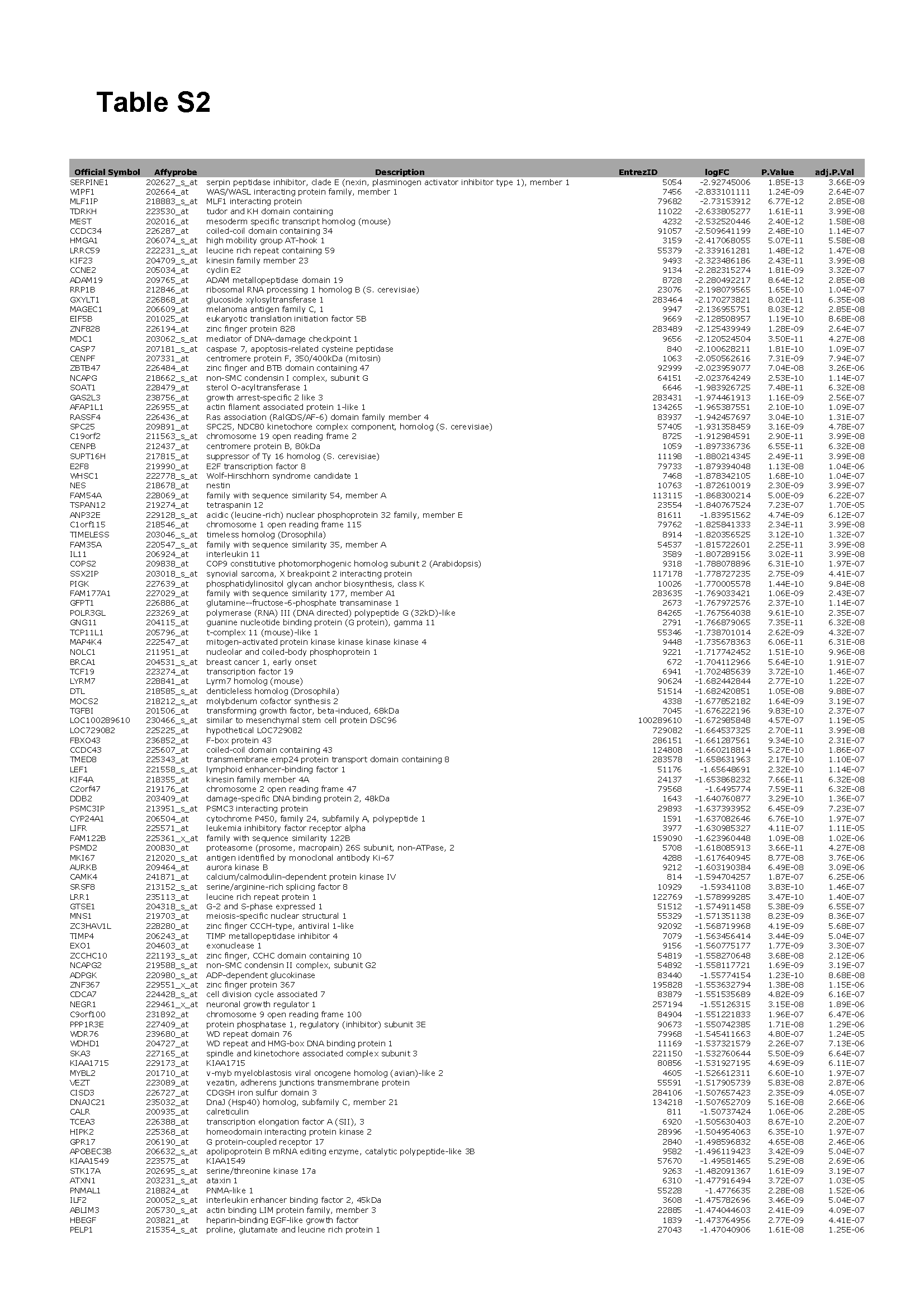


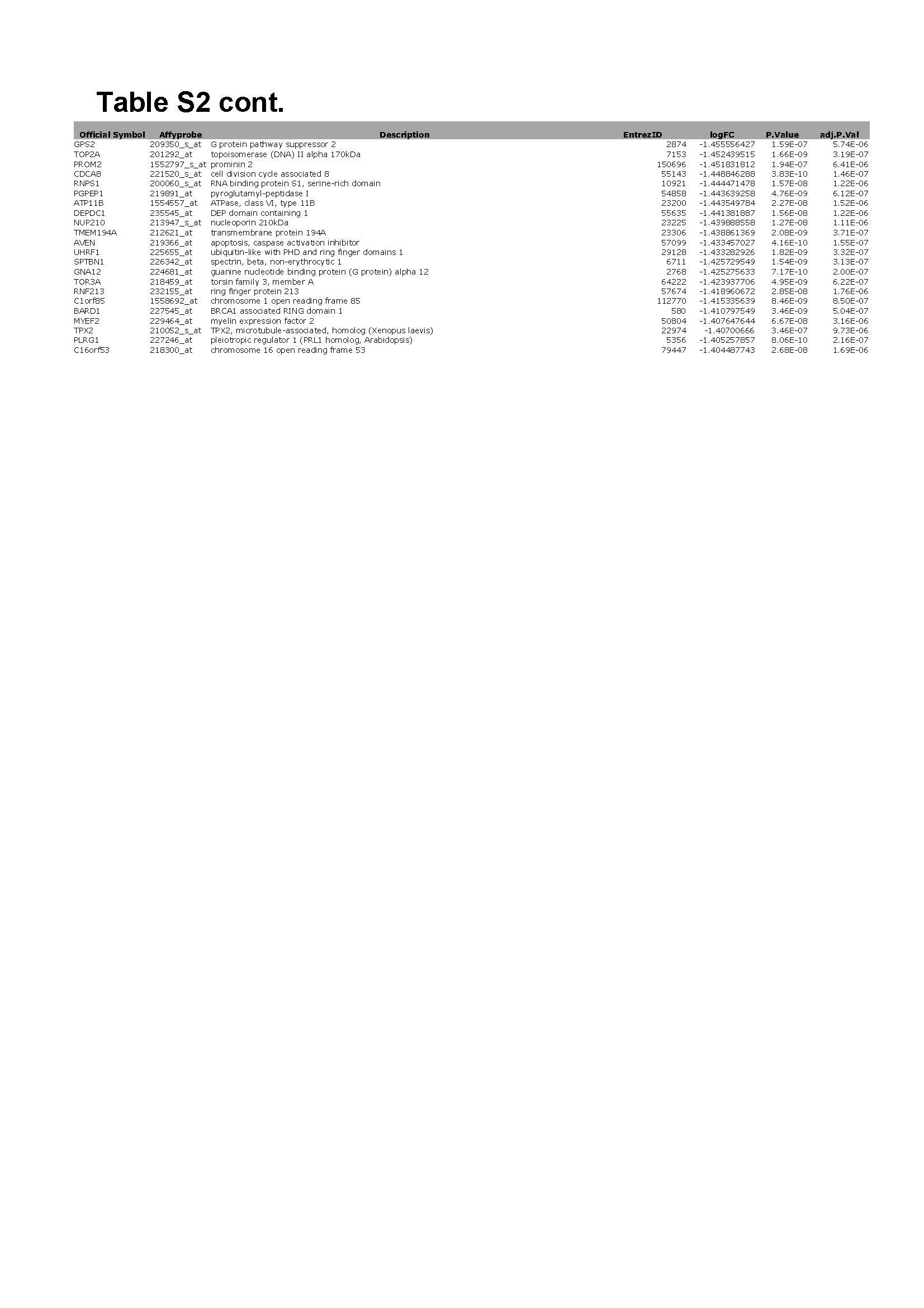


Table S3. Functional analysis

S3A
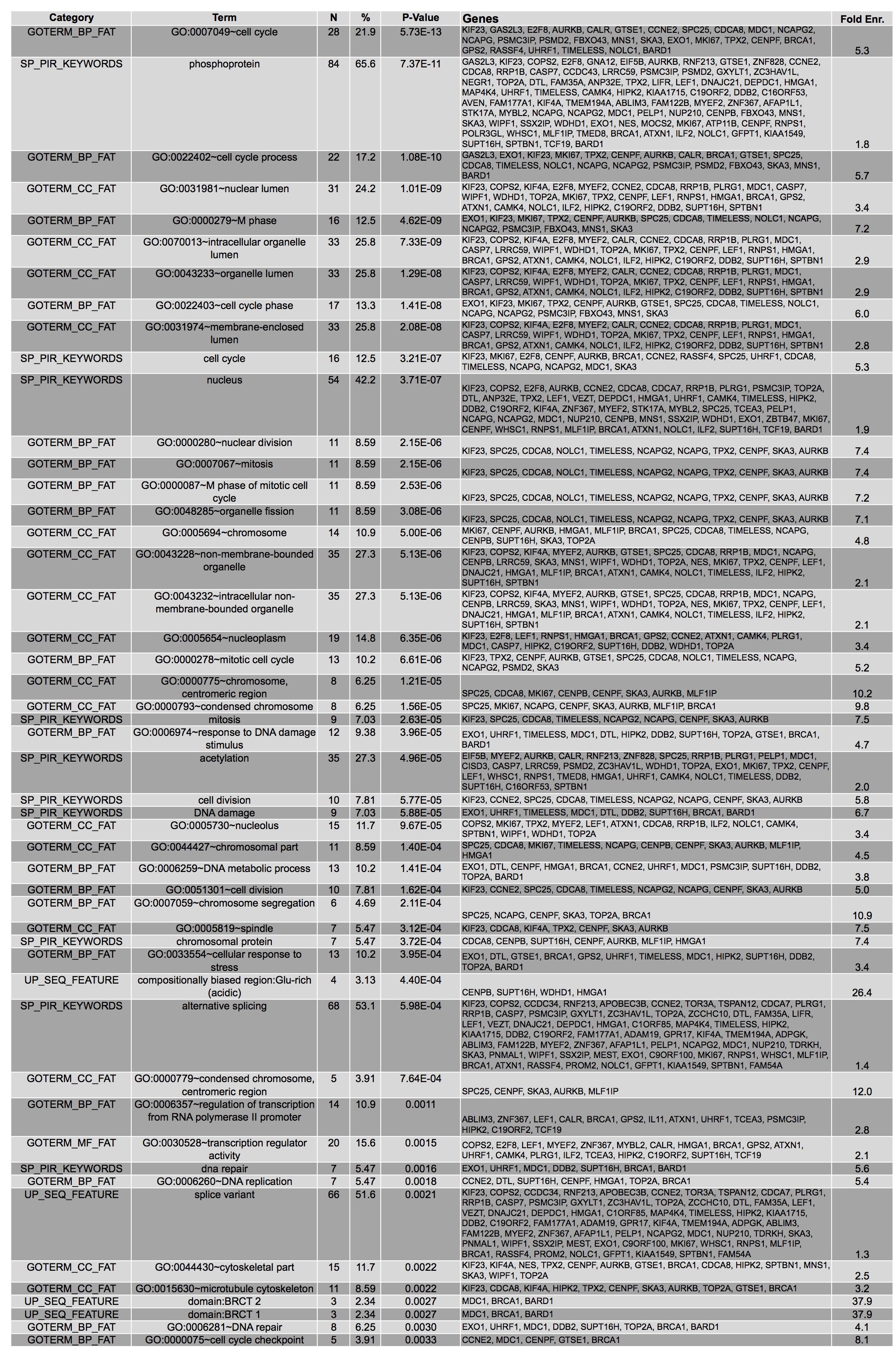

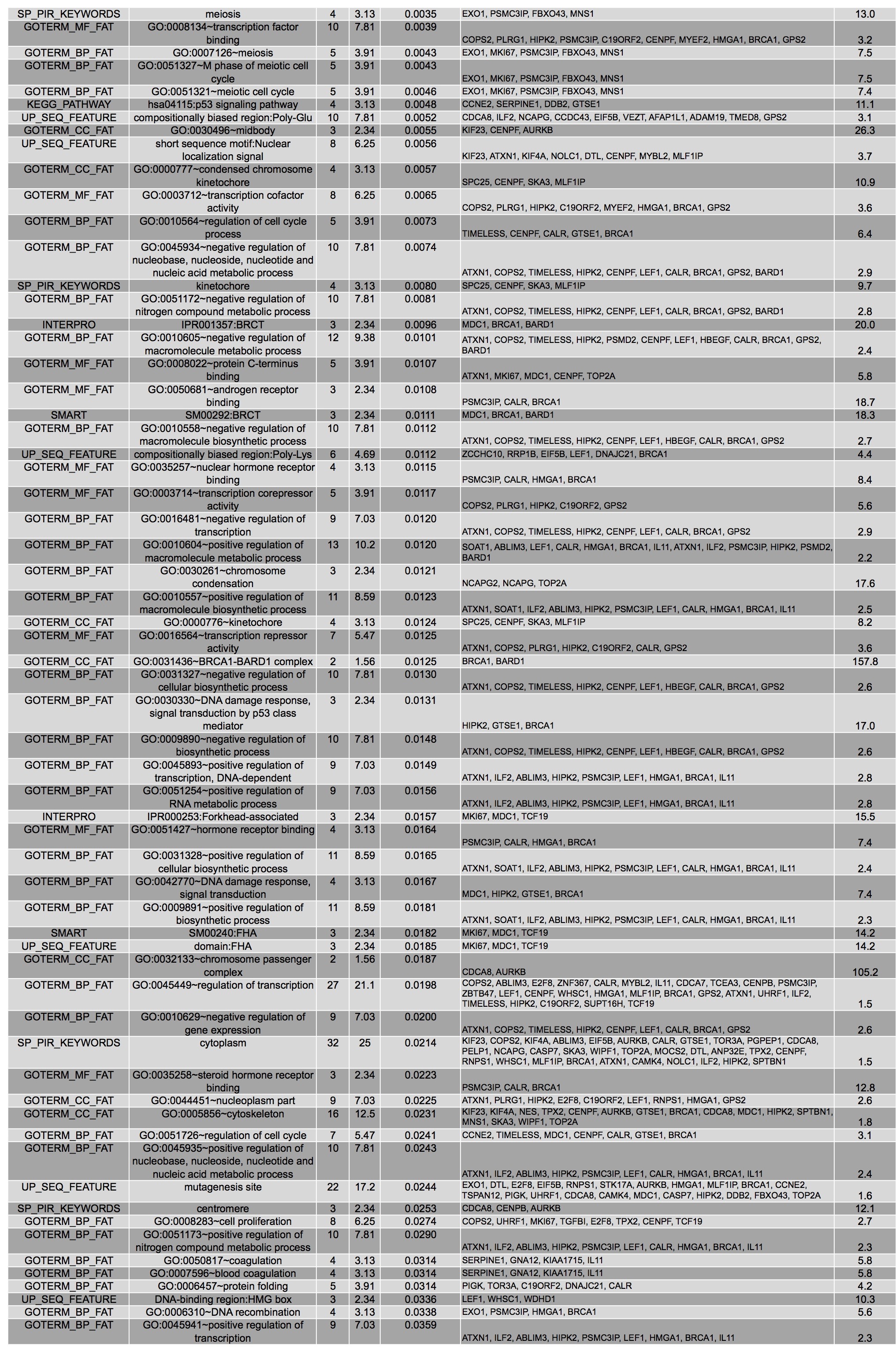

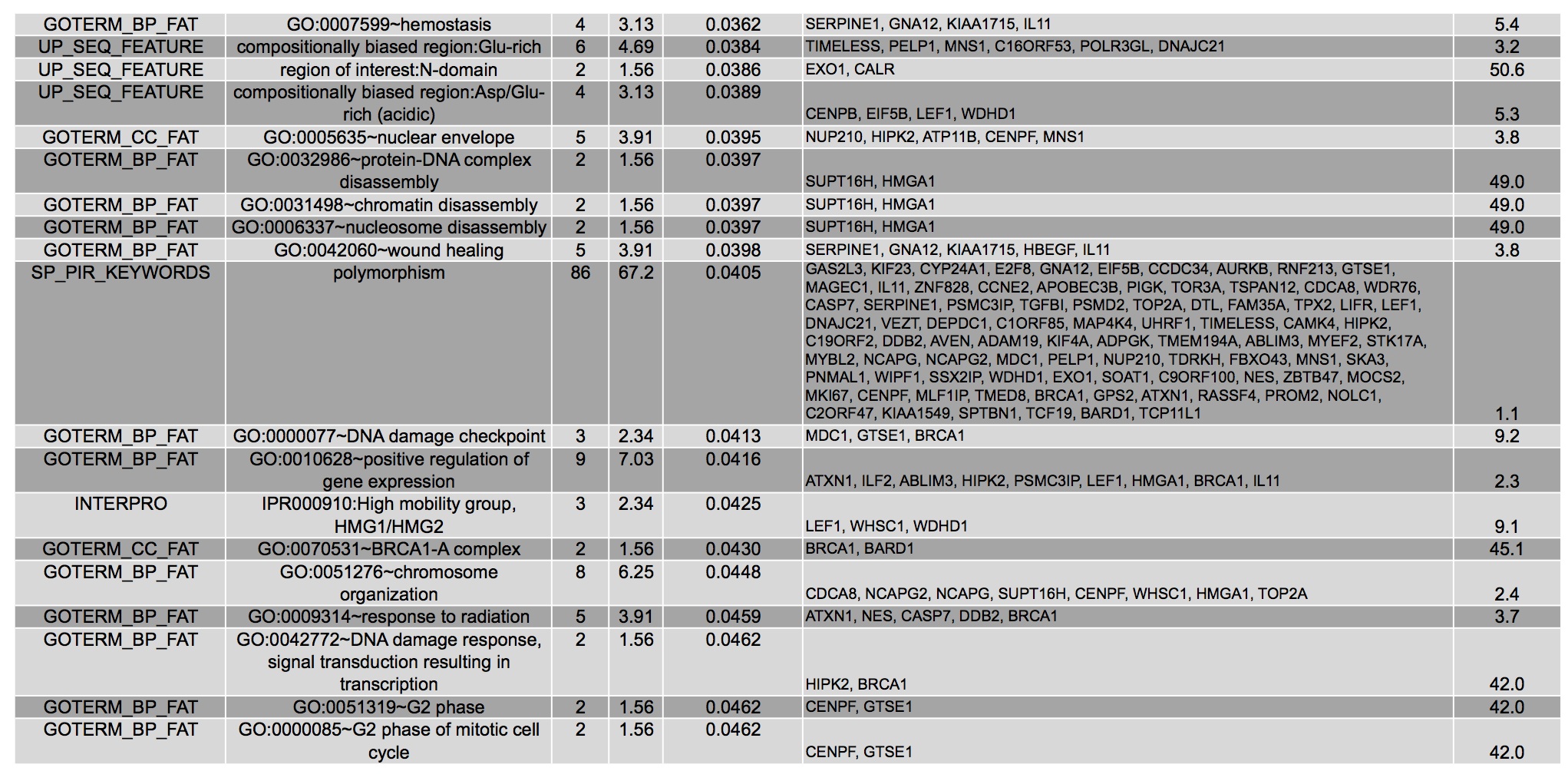


S3B


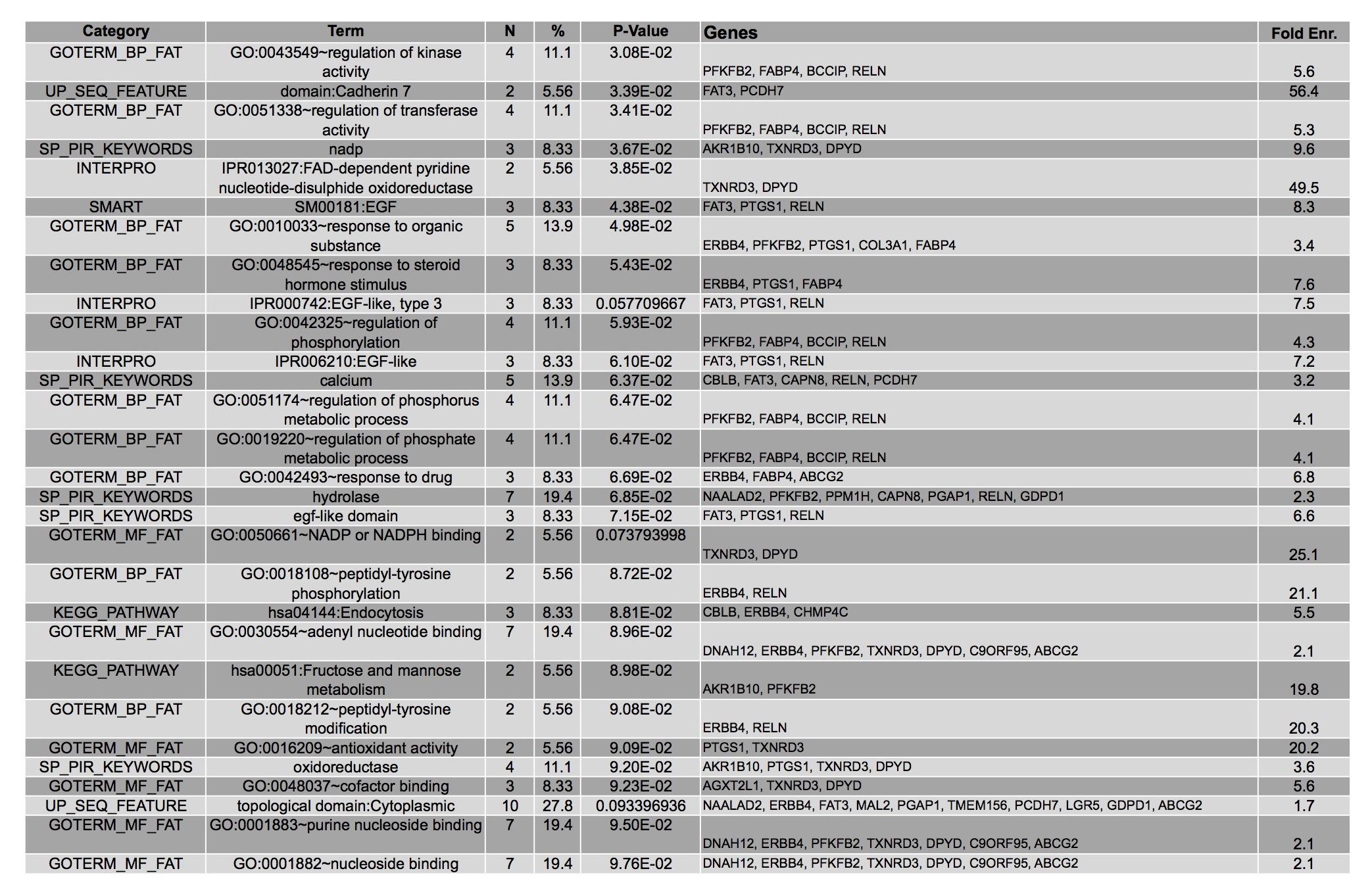


Table S4. Multivariate analysis


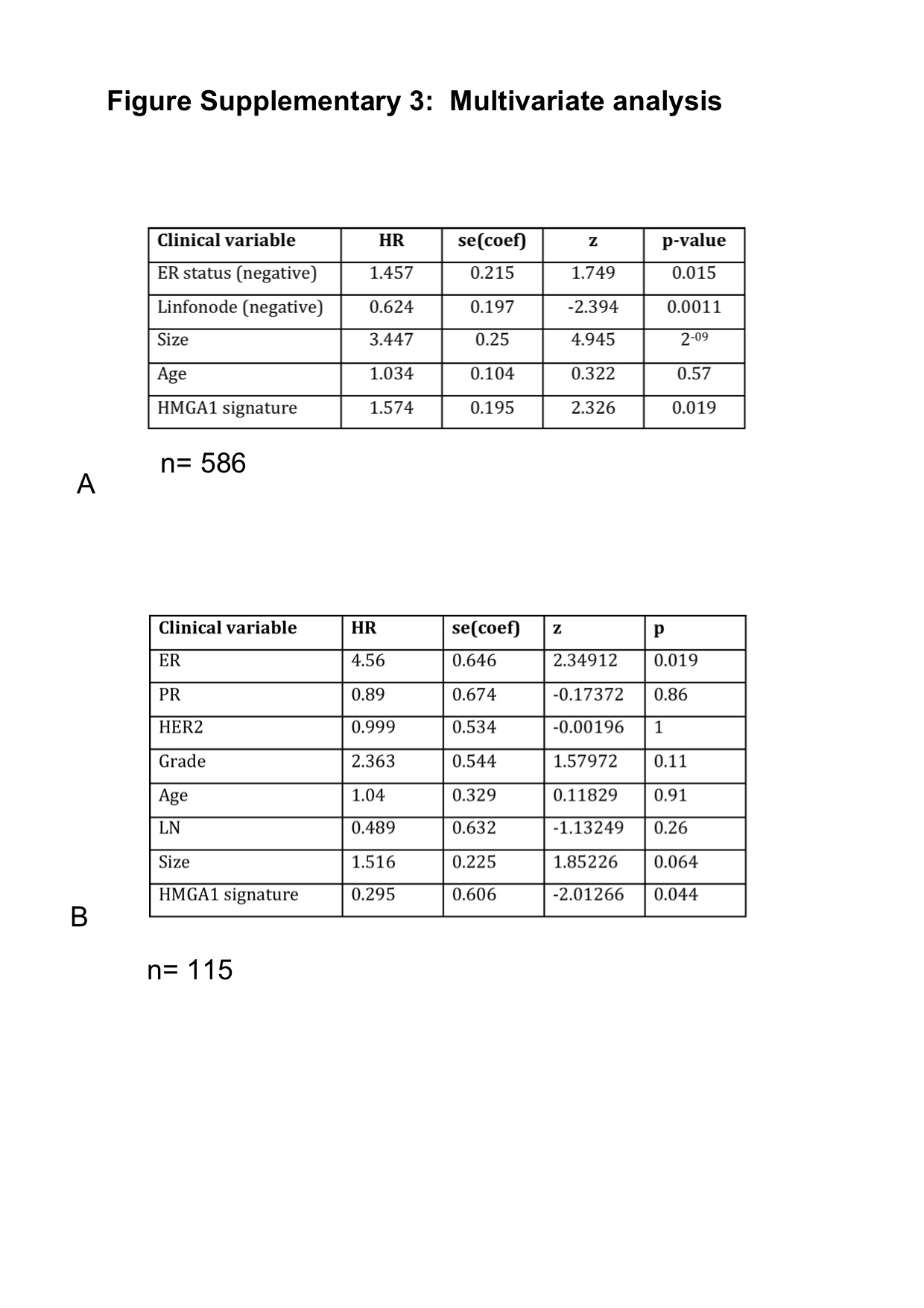


SUPPLEMENTARY TABLE LEGENDS

Tables S1 and S2. Differentially expressed genes in the MDA-MB-231 breast cancer cell model. Starting from the global gene expression matrix of the gene expression profiles of the human MDA-MB-231 cell lines with HMGA1 depletion and the control cells, differential gene expression was performed with the R/Bioconductor environment limma package. We selected the genes that had expression in the silenced HMGA1 cells greater or lower than a 1.4 log fold change relative to the control cells (Table S1 and Table S2, respectively).

Tables S3. Functional annotation of the HMGA1-modulated genes. Starting from the genes that had expression in silenced HMGA1 cells lower (A) than a 1.4 log fold change or greater (B) than a -1.4 log fold change with respect to the control cells, we used the publicly accessible software DAVID/EASE with default parameters. The complete GO term list is presented.

Tables S4. Multivariate analysis of the risk of death. Multivariate analysis was performed on the same cohort of patients analyzed in Figure 1 using the Cox proportional hazards regression modeling. We searched for the relationships between the high HMGA1 signature and other variables used in the clinical practice. Our analysis indicated that the HMGA1 signature behaves as an independent predictor of poor clinical outcome. The multivariate analysis has been done on 115 samples with the corresponding clinical variables.

SUPPLEMENTARY METHODS

*In vivo* Experiments

For the intravenous injection, 100,000 cells were resuspended in 200 µl of DMEM for each mouse, whereas for the fat pad injection, 1 million of cells were resuspended in 100 µl of DMEM. We performed *in vivo* imaging at 20 and 26 days after i.v. injection or at 14, 21, 28, 34 and 40 days after fat pad injection. Anesthetized animals (1-3% isoflurane, Merial Italia S.p.A, Italy) were given the substrate D-Luciferin (Biosynth AG, Switzerland) by intraperitoneal injection at 150 mg/kg in PBS (Sigma). Imaging times ranged from 15 seconds to 5 minutes, depending on the tumor model and time point. The light emitted from the bioluminescent tumors or metastasis was detected using a cooled charge-coupled device camera mounted on a light-tight specimen box (IVIS Lumina II Imaging System; Caliper Life Sciences, Alameda, CA). Regions of interest from the displayed images were identified around the tumor sites or metastasis regions, such as the lymph node and lungs, and quantified as total photon counts (photon/s) using Living Image® software (Xenogen). In some experiments, the lower portion of each animal was shielded before reimaging to minimize the bioluminescence from the primary tumor to ensure that the signals from the metastatic regions could be observed *in vivo*. For *ex vivo* imaging, 150 mg/kg of D-Luciferin was injected into the mice just before necropsy. The lungs were excised and imaged for 5 minutes.

Cluster Analysis

Starting from the normalized annotated expression matrix after gene median centering, features that had standard deviation of less than 0.3 were filtered out. Unsupervised hierarchical cluster analysis (average-linkage method) was performed using Cluster software (EisenLab). Cluster results were then visualized using Java TreeView.

Functional Analysis

Differentially expressed gene lists obtained from low-level procedures were analyzed for functional associations.

- Data were analyzed through DAVID Bioinformatics Resources v6.7 (49) using the suggested standard parameters.
- Data were analyzed through the Oncomine Pro web tool using suggested standard parameters. Custom concept analysis was performed, and the “Summary view” (adapted) was reported.
- Data were analyzed through Ingenuity Pathway Analysis (IPA) software. Core analysis was performed, and the top associated networks table was reported.
